# Urban expansion simulation with an explainable ensemble deep learning framework

**DOI:** 10.1016/j.heliyon.2024.e28318

**Published:** 2024-03-22

**Authors:** Yue Zhu, Christian Geiß, Emily So, Ronita Bardhan, Hannes Taubenböck, Ying Jin

**Affiliations:** a Swiss Federal Institute of Technology, ETH Zurich, Department of Civil, Environmental and Geomatic Engineering, Institute of Environmental Engineering, Hydrology and Water Resources Management, Laura-Hezner-Weg 7, 8093, Zurich, Switzerland; b The Department of Architecture, University of Cambridge, CB2 1TN, Cambridge, UK; c The German Remote Sensing Data Center (DFD), German Aerospace Center (DLR), 82234, Weßling, Oberpfaffenhofen, German

**Keywords:** Urban expansion simulation, Deep learning, Machine learning, Ensemble framework, Spatial modeling

## Abstract

Urban expansion simulation is of significant importance to land management and policymaking. Advances in deep learning facilitate capturing and anticipating urban land dynamics with state-of-the-art accuracy properties. In this context, a novel deep learning-based ensemble framework was proposed for urban expansion simulation at an intra-urban granular level. The ensemble framework comprises i) multiple deep learning models as encoders, using transformers for encoding multi-temporal spatial features and convolutional layers for processing single-temporal spatial features, ii) a tailored channel-wise attention module to address the challenge of limited interpretability in deep learning methods. The channel attention module enables the examination of the rationality of feature importance, thereby establishing confidence in the simulated results. The proposed method accurately anticipated urban expansion in Shenzhen, China, and it outperformed all the baseline methods in terms of both spatial accuracy and temporal consistency.

## 1 Introduction

### 1.1 Motivation

Along with the growth of the global population, urban land has been expanding at a very high rate [1]. Inadequately planned or poorly managed urban expansion are very likely to result in uneven growth rates and unsustainable developments [2], which can further lead to unintended severe consequences, such as increasing the vulnerability to natural hazards [3]. In this sense, methods that can simulate the trend of urban expansion or predict the changes of future land use distribution are critical for sustainable land management and urban planning [4].

Since urban areas are complex and multidimensional systems that are influenced by a great variety of factors [5], one of the major challenges is discovering hidden patterns from large databases [6]. Traditional rule-based methods were being criticized in terms of the efficiency of tackling intricate variables relationships [7], as well as the generalizability to areas that follow bespoke rules [8]. Unlike conventional rule-based methods that have rigid intelligence heavily dependent on fixed man-defined rules, Deep Learning (DL) methods excel in learning complex non-linear relationships from data with its high-dimensional feature space. Also, land change data is in essence spatio-temporal data, whereas traditional Cellular automata (CA) based methods are generally incapable of considering long-term temporal dependency, and this issue has received little attention in existing research on urban land change modelling [9]. Arguably, considering long-term relationships is one of the advantages of sequential DL models (e.g., RNNs, Transformers).

However, the solution paths of DL models are difficult to trace and explain due to a frequently very high degree of nonlinearity, and the attribute of difficulty to explain significantly constrained their implementation in many fields [10]. Especially for the fields that involve critical decision making, such as urban planning, the ability to understand the key contributors to model results is critical information to support policymaking.

Thereby, we propose a novel ensemble framework that not only leverages DL techniques to accurately predict fine-grained urban land changes, but also provides an interpretable mechanism for gaining confidence in the simulated results. The simulation method is developed for simulating spatio-temporal changes in land-use and land-cover (LULC) patterns, which is crucial for understanding the urbanization process and supporting sensible decision-making [11].

### 1.2 Existing models for land change simulation

The importance of spatially-explicit models for studying and predicting land dynamics has been increasingly recognized [12]. CA-based methods and neural network (NN) based methods are two main types of solutions for LULC simulation that account for spatial relationships.

Regarding CA methods, SLEUTH [8] and CLUE-S [13] are well-known methods for predicting land changes [[14], [15], [16]]. Since CA methods are generally based on defined rules that heavily rely on experience and expertise, bias is likely to be implicit in CA simulations [17], especially considering the relationships between the driving factors of land dynamics are often highly non-linear. The cells in CA models can be regarded as an abstraction of actual land use maps [18] in terms of spatial resolution and thematic classes. Although such simplicity can bring efficiency, the compromised resolution can also result in a loss of important details.

NN-based methods have been applied in various fields related to urban growth studies, including providing a solution for LULC simulation [[19], [20], [21], [22], [23]]. Pijanowski et al. [22] proposed a GIS-based ANN method using spatial factors as model inputs to predict land changes. Li and Yeh (2002) integrated an ANN with a CA method to simulate LULC changes with various spatial variables. Zhou et al. [24] coupled a deep belief network with CA. Also, Wang et al. (2018) proposed an ANN model which included historical LULC data and multiple geographical features as input to anticipate rapid LULC changes.

Although ANNs showed superior learning capacities for complex non-linear tasks compared with conventional rule-based methods [25,26], they have been criticized for their “black box” properties, which leads to difficulties in explaining and ranking the importance of variables (Dreiseitl and Ohno-Machado, 2002). More critically, conventional ANNs lack the capability of capturing spatial or temporal dependency in the data.

### 1.3 Deep learning models for spatio-temporal tasks

Advances in computation capacities and the release of big data have significantly empowered the implementations of Deep Neural Networks (DNNs), which generally contain far more hidden layers that form a much deeper architecture than traditional shallow ANNs. Consequently, DNNs are more effective in mining a large amount of data [7], analyzing complex non-linear features with higher accuracy, as well as capturing the dynamic complexity of LULC change [18].

Two types of DNN methods, Convolutional Neural Networks (CNNs) and Recurrent Neural Networks (RNNs) are developed for processing spatial and sequential data respectively. CNNs showed outstanding performance on a two-dimensional image topology [27]. They can extract high-dimensional key features from image data through conducting convolutional computation with multiple sliding windows. For example, Qian et al. [28] adopted a 3D CNN to extract spatiotemporal neighborhood features for simulating land use change. As subsets of RNNs, Long-short Term Memory (LSTM) [29] and Gated Recurrent Unit (GRU) [30] were proposed with gates mechanism enables them to become more effective and scalable in maintaining long-term states [31]. Also, LSTM and GRU were developed with CNNs to better handle spatial-temporal data. Regarding their applications related to land change simulations, Pan et al. [32] adopted the weighted sum of the outputs of an U-Net and a LSTM for urban expansion simulation. Ma et al. [33] compared the accuracy of a FC-LSTM and a ConvLSTM on projecting vegetation changes based on spatio-temporal NDVI values. Geng et al. [34] coupled a 3D-CNN with CA to learn latent spatio-temporal dependency for LULC change simulation.

More recently, the advanced performance of transformer-based models has been widely recognized in various fields, including both language tasks [35] and vision tasks [36]. Transformers were suggested to have even better ability than CNNs in terms of preserving spatial information (Raghu et al., 2021). Since transformers yielded excellent performance for both sequential data processing and spatial feature extraction, they tend to have the potential in facilitating new LULC simulation frameworks dealing with spatial-temporal data inputs.

This study proposes a novel DL-based ensemble framework for urban expansion simulation at an intra-urban granular level. The study is based on two assumptions: first, that time series LULC data reflect the trajectory of previous LULC changes. Second, spatio-temporal DL methods can extract such trajectory features and leverage them to project future spatio-temporal heterogeneous changes in LULC patterns. Since modelling sequential patterns with mixed types of input data can generate redundant information and harm model efficiency (Muckley & Garforth, 2021), processing dynamic variables (e.g., housing price) and stationary variables (e.g., slope) together in urban expansion simulations is very likely to compromise model performance. Thus, we intend to develop a tailored mechanism that can differentiate constant variables and sequential variables. The main contributions can be summarized as follows:

(i) investigates the capability of DL methods in learning spatio-temporal heterogeneous features for LULC simulation at an intra-urban granular level;
(ii) proposes a transformer-based framework that is tailored for urban expansion simulation with superior simulation accuracy;
(iii) studies on developing interpretable DL methods for LULC simulation, the study provides a method for gaining confidence in the simulated LULC changes through examining the rationality of the feature importance.

The remainder of this manuscript is organized in the following manner: Section 2 presents the proposed methodology for urban expansion simulation. Section 3 introduces the data set and the experimental setup. Section 4 and Section 5 report the discussion of the experiment results and the conclusions, respectively.

## 2 Study area and materials

An urban area of 1296 square kilometers in Shenzhen was selected as the study area, as the city has witnessed a dramatic urban expansion over the past four decades [37]. During the process of urbanization, both expansions from non-urban to urban and demolished old settlements for urban regeneration were witnessed in Shenzhen. Empirical studies suggested that the urban growth in Shenzhen shows a consistent pattern that follows the theory of path dependency [38]. In this sense, the consistency of spatial and temporal changes in the LULC patterns of Shenzhen has the potential to facilitate the simulation and prediction of future LULC patterns. As such, the characteristic of the rapid development of Shenzhen renders it a suitable case study for this study. Further, although Shenzhen has some unique characteristics (e.g., strategic political position), it also shares many characteristics with other cities that are undergoing rapid urbanization processes (e.g., encroachment on vegetation coverage). Consequently, taking Shenzhen as an example to develop a tool for simulating and forecasting LULC changes can bring benefits to policymaking related to urban land dynamics.

**Fig. 1:**
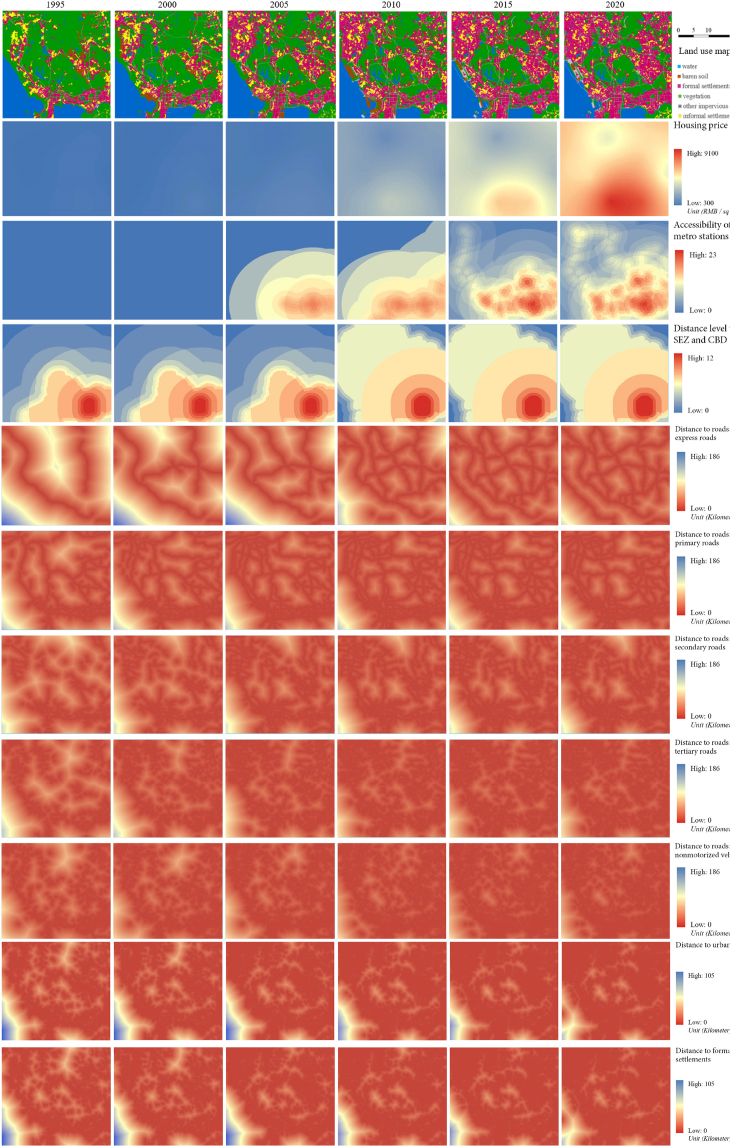
The multi-temporal predictor variables for LULC prediction.

The data of LULC maps contains six time steps from 1995 to 2020 with an interval of approximately 5 years (Fig. 1). The multi-temporal LULC maps employed were generated by a U-Net-ConvLSTM model with a post-classification relearning strategy [39] based on Landsat remote sensing images. The classification accuracy reaches 84.9% on a validation dataset. Misclassified pixels and seasonal variations also calibrated based on available satellite images. The LULC category adopted in this study contains six thematic classes, including water, vegetation, barren soil, other impervious surfaces, formal settlements, and informal settlements.

**Fig. 2:**
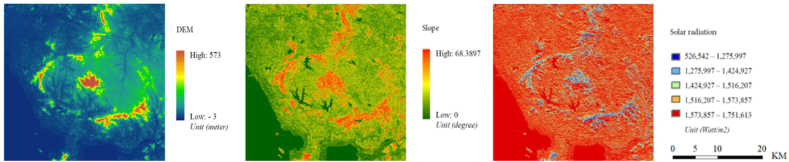
Static variables for LULC prediction.

Besides multi-temporal LULC maps, a series of predictor variables were adopted as inputs for simulating urban expansion, including (1) previous multi-temporal land use maps, (2) distribution of housing price, (3) distance to metro stations, (4) distance to the Special Economic Zone (SEZ) and the Central Business District (CBD), (5) Digital Elevation Model (DEM), (6) slope, (7) solar radiation, (8) distance to roads, and (9) distance to urban extents. These predictor variables are grouped into multi-temporal variables and static variables. Static variables mainly refer to the variables that do not change substantially over decades, such as DEM, slope and solar radiation (Fig. 2), whereas multi-temporal variables included social-economic variables and infrastructural variables, such as housing price, distance to roads, accessibility of metro stations, and distance to the SEZ and the CBD. All the predictor variables were standardized and stacked with one-hot pre-processed land use maps to become the input of the tested models.

## 3 Methods

### 3.1 Model construction

#### 3.1.1 Ensemble framework

**Fig. 3:**
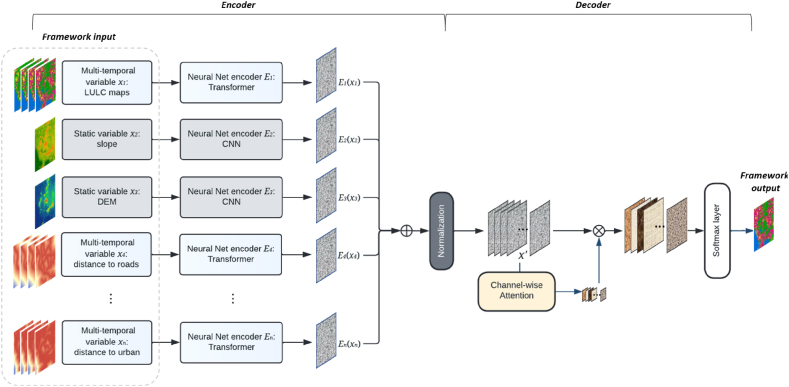
Overall structure of the proposed ensemble framework.

The proposed method adopted an ensemble framework consists of an encoder and a decoder (Fig. 3). Ensemble learning refers to an approach of combining multiple models for making a decision, and each model in an ensemble framework can be regarded as a base learner [40]. It has been widely recognized that the performance of an ensemble method is generally better than the performance of an individual learner [41]. Even based on the same settings and identical model architectures, the stochastic essence of learning-based methods often results in slightly or substantially different mapping functions from input to output. The upside of this effect is that different models will not always make the same false predictions on the test data set [27]. This leads to the basic premise of ensemble learning, which is the collective decisions of a set of learners could compensate for the errors produced by a single base learner, thereby contributing to better overall performance [40].

Regarding the encoder, it can be considered as a multi-model structure consisting of multiple neural networks, among which a transformer-based model was developed as the main backbone model for encoding spatio-temporal variables. Different types of input variables (i.e., static variables and dynamic variables) are treated differently to avoid redundant parameters and achieve efficiency gains. Although ensemble methods often achieve better performance than a single network [41], large ensembles of very deep models could lead to high computational costs [42]. To reduce computational costs, we deployed vanilla 2D Convolutional Neural Networks (CNNs) to process static spatial variables (e.g., slope, DEM, and solar radiation). With it, we decrease the whole size of the ensemble framework. Regarding the multi-temporal features, transformer-based networks were developed to encode multi-temporal spatial features (e.g., land use maps, distance to roads).

Each set of variables was encoded through an independent pipeline. Then each encoded feature was normalized before they were fed for decoding. The output of the encoder $x^{\prime }$ can be regarded as a normalized assembly of encoded variables ($E_{1}\left(x_{1}\right),E_{2}\left(x_{2}\right),\ldots ,E_{n}\left(x_{n}\right)$) through each pipeline. It can be expressed as:

(1) $$x^{\prime }=Norm\left(E_{i}\left(x_{i}\right)\right)=Norm\left(E_{1}\left(x_{1}\right),E_{2}\left(x_{2}\right),\ldots ,E_{n}\left(x_{n}\right)\right)$$

where $x_{i}$ represents each input variable for urban change simulation, $E_{i}\left(x_{i}\right)$ refers to the encoded features produced by each neural net encoder $E_{i}$.

The decoder contains a channel attention layer to excite important channels for simulation, as well as to indicate the main contributors to model output. All the encoded features are stacked together with the generation of a new dimension, which can be regarded as the channel dimension. Then Batch Normalization [43] is adopted as the normalization method to adjust the means and variances of the values in each layer. Following such transformation, a pair of learnable parameters γ and β are applied for scaling and shifting the normalized feature to help the network maintain its representation power. Details of the application normalization function ${BN}_{\gamma ,\beta }$ on the encoded features $E_{i}\left(x_{i}\right)$ can be expressed as below:

(2) $$\mu =\frac{1}{m}\sum_{i=1}^{m}E_{i}\left(x_{i}\right)$$

(3) $$\sigma^{2}=\frac{1}{m}\sum_{i=1}^{m}{\left(E_{i}\left(x_{i}\right)-\mu \right)}^{2}$$

(4) $$x^{\prime }={BN}_{\gamma ,\beta }\left(E_{i}\left(x_{i}\right)\right)=\gamma \frac{E_{i}\left(x_{i}\right)-\mu }{\sqrt{\sigma^{2}+\epsilon }}+\beta$$

where $\sigma^{2}$ and μ respectively represents the variance and mean of each batch. ϵ is a constant for achieving numerical stability, m refers to batch size. The normalized features $x^{\prime }$ are then fed into a channel-wise attention layer to achieve the excitation of channel-wise information. Subsequently, the channel-wise recalibrated features $x^{\prime \prime }$ are passed through a Softmax activation layer for the generation of the final output Y:

(5) $$Y=Softmax\left(x^{\prime \prime }\right)=Softmax\left(W_{channel}⊗x^{\prime }\right)$$

where ⊗ denotes element-wise multiplication.

#### 3.1.2 Transformer-based models for spatial pattern modelling

**Fig. 4:**
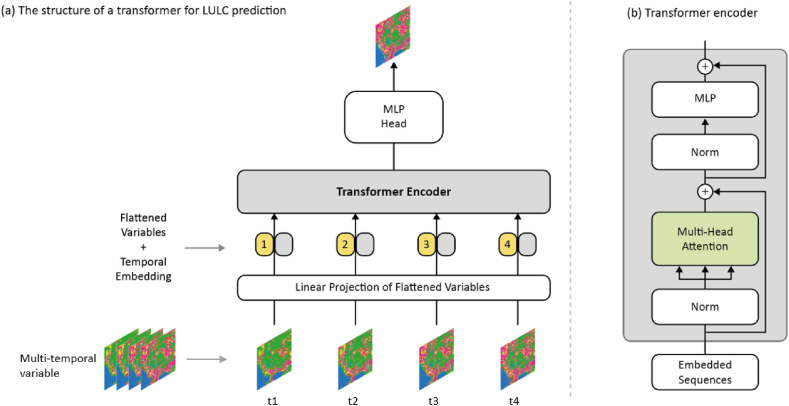
The architecture of the transformer-based backbone model.

The structure of the transformer-based encoder was developed based on the Vision Transformer (ViT) [36] (Fig. 4). The signature feature of ViT is that it contains alternating layers of multi-headed self-attention blocks. To feed image data into a ViT, images need to be divided into small patches along the spatial dimension and flattened into linear sequences. Then the positional information of each split patch is added into the input sequence using position embedding, which can store the positional information with a representative value that acted as a positional code [36]. As such, the positional relationship of the small patches can be recognized during the training process. In the proposed framework, the input sequences were split along the temporal dimension, thus temporal information can be encoded into the input sequence for model training.

Transformer encoders contain alternating layers of multi-headed self-attention blocks, which consist of multiple scaled dot-product attention modules [35,36]. For each scaled dot-product module, its input consists of queries Q, keys K, and values V, which are generated by applying a fully connected layer on the normalized input value $x_{i}$. The normalization method employed in this transformer encoder is Layer Normalization [44], which normalizes values across the channel dimension and is not constrained by certain batch sizes. The values of Q, K, and V are forwarded to scaled dot-product attention modules to get self-attention weights. The attention function of a scaled dot-product attention module is computed as:

(6) $$Attention\left(Q,K,V\right)=softmax\left(\frac{QK^{T}}{\sqrt{d_{k}}}\right)V$$

where

(7) $$Q=LayerNorm\left(x_{i}\right)W_{i}^{q}$$

(8) $$K=LayerNorm\left(x_{i}\right)W_{i}^{k}$$

(9) $$V=LayerNorm\left(x_{i}\right)W_{i}^{v}$$

where $d_{k}$ refers to the dimension of queries and keys, $\sqrt{d_{k}}$ is a scaling factor. Then SoftMax function is employed to get the attention weights. After that, the outputs of all the scaled dot-product modules are concatenated together to feed into a fully connected layer to form a multi-head attention module:

The multi-head attention module is followed by a feed-forward multi-layer perception (MLP) module, which contains two linear layers and adopts GELU as the activation function:

(10) $$MultiHead\left(Q,K,V\right)=Concat\left({head}_{1},\ldots ,{head}_{h}\right)W^{o}$$

where

(11) $${head}_{i}=Attention\left(QW_{i}^{hq},KW_{i}^{hk},VW_{i}^{kv}\right)\text{.}$$

(12) $$MLP\left(z_{l}\right)=\left(GELU\left(z_{l}W^{l}+b^{l}\right)\right)W^{l+1}+b^{l+1}$$

where

(13) $$z_{l}=LayerNorm\left(MultiHead\left(Q,K,V\right)+x_{i}\right)$$

(14) $$t=MLP\left(z_{l}\right)+z_{l}$$

where $z_{l}$ represents the output of MultiHead(Q,K,V), $b_{l}$ refers to the bias in each linear layer. Residual connections are set up after each block, as can be found in Eq. (12) and Eq. (13). t is the output of the transformer encoder. Then a MLP head module is set as the last layer to generate the final output of the transformer encoder $E_{i}\left(x_{i}\right)$:

(15) $$E_{i}\left(x_{i}\right)={MLP}^{\prime }\left(t\right)=LayerNorm\left(t\right)W^{t}+b^{t}$$

The encoded features $E_{i}\left(x_{i}\right)$ are synthesized for the next stage of computation following the equations introduced in Section 3.1.

#### 3.1.3 Interpretation through channel attention and feature importance

As discussed in Section 1.1, although neural network-based models demonstrate superior performance in terms of accuracy and efficiency, they are often criticized for being difficult to interpret. To address this issue, we intend to incorporate a tailored module into the model structure to provide insights into the importance of each input feature. Specifically, this interpretable module is based on the structure of a channel-wise attention mechanism.

Attention mechanisms were widely utilized in DL models to achieve performance gains [[45], [46], [47]]. These mechanisms operate using trainable weights, which enhance the sensitivity of DL models to useful features and reduce attention to less important information. Channel attention mechanisms, in particular, focus on recalibrating features to emphasize important channels. In our proposed ensemble framework, since each channel of the input layers corresponds to an input feature, the weights of these channels can be interpreted as indicating the relative importance of each input feature.

**Fig. 5:**
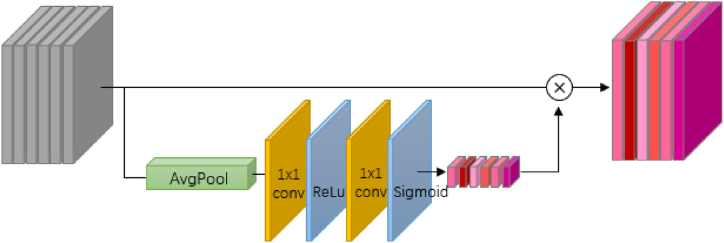
Architecture of the channel attention module.

The channel-wise attention module deployed in this study followed the Squeeze-and-Excitation Network (SE-Net), which is a computationally lightweight structure that utilizes global information for channel-wise excitation [48]. The SE network consists of a global average pooling layer, convolutional layers, following with activation layers (Fig. 5), which can be expressed as below:

(16) $$W_{channel}=M_{c}\left(x^{\prime }\right)=\sigma \left(f^{1\times 1}\left(ReLU\left(f^{1\times 1}\left(AvgPool\left(x^{\prime }\right)\right)\right)\right)\right)$$

where σ refers to Sigmoid activation function.

Although the channel attention mechanism can treat channels discriminatively, their main function is feature calibration rather than an explicit indication of feature importance. Also, the channel attention blocks are mainly effective in the position where it is applied to. For instance, the implementation of channel attention blocks at a shallow-depth location can excite lower-dimensional features, and the implementation at a deep-depth location is more associated with output classes [48]. In order to indicate feature importance, we exploited the channel attention module in a tailored manner in the proposed ensemble framework. To be more specific, each predictor variable is encoded through an independent pipeline before being fed into the channel attention block, which is implemented in the last part of the framework. In this manner, the channel-wise weights can reveal the contribution of the high-dimensional representation of each predictor variable to the simulated results.

### 3.2 Simulation of urban expansion

#### 3.2.1 Experiment setup

**Fig. 6:**
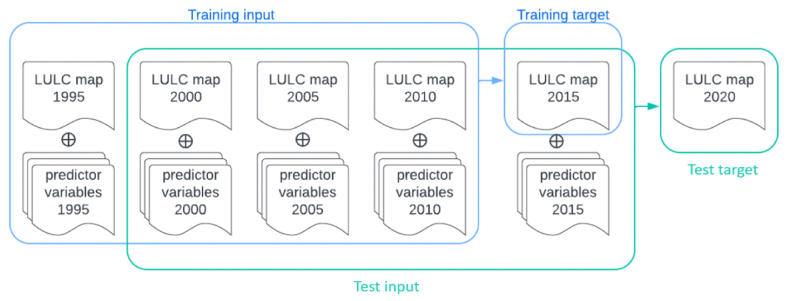
Experiment setup for LULC prediction.

The multi-temporal data sets were divided along the temporal dimension into a training dataset and a test dataset. The training data set contains the first five time steps (1995–2015), whereas the test data set consists of the last five time steps (2000–2020). In both train and test datasets, the first four-time-step variables were adopted as input and the last time step was used as the training and validation labels. The size of each variable in spatial dimension is 1200 by 1200 pixels. The training data set was spatially subsampled into 200 pieces of small images, each measuring 256 by 256 pixels. 35 pieces of these subsampled images of the training set were used for validation during the training process. The evaluation on test dataset was conducted on the entire spatial coverage of the simulated land use map for the year 2020 (Fig. 6).

Four baseline models were employed to evaluate the proposed methods, including a MLP, a Convolutional Gate Recurrent Unit (ConvGRU), a transformer-based model that was developed based on a ViT, and an ensemble framework that employed ConvGRU as the backbone model, named Ensemble ConvGRU. It should be noted that the settings of the MLP model are based on the Land Transformation Model, which is a well-known ANN model adopted vector data as input for urban expansion simulation [22], thus it was trained with single-temporal data. Besides comparing the proposed method with four neural network-based baseline models, we conducted a comparison with a well-accepted CA-based land change simulation model, SLEUTH [8].

All the tested methods were trained for 100 epochs with the following settings: the batch size is 4 and the learning rate is 8 × 10^-4, the adopted optimizer and loss function is Adam and cross-entropy loss.

#### 3.2.2 Performance evaluation

A series of evaluation metrics were employed to evaluate the accuracy of simulation results generated by all the tested methods, the metrics include (i) overall accuracy assessment metrics (4i.e., kappa statistics [49,50], F1, Precision, and ROC-AUC); (ii) the quantity and allocation disagreement [51]; (iii) spatial distribution of correctness and errors [52,53].

Additionally, the simulation results were also evaluated from the perspective of urban morphology. Five different landscape indices were employed, including (i) Total Area (TA/CA), (ii) Largest Patch Index (LPI), (iii) Landscape Shape Index (LSI), (iv) Mean Euclidean Nearest Neighbor Distance (ENN_MN), and (v) Aggregation Index (AI).

## 4 Results

### 4.1 Visual inspection of the simulation results

**Fig. 7:**
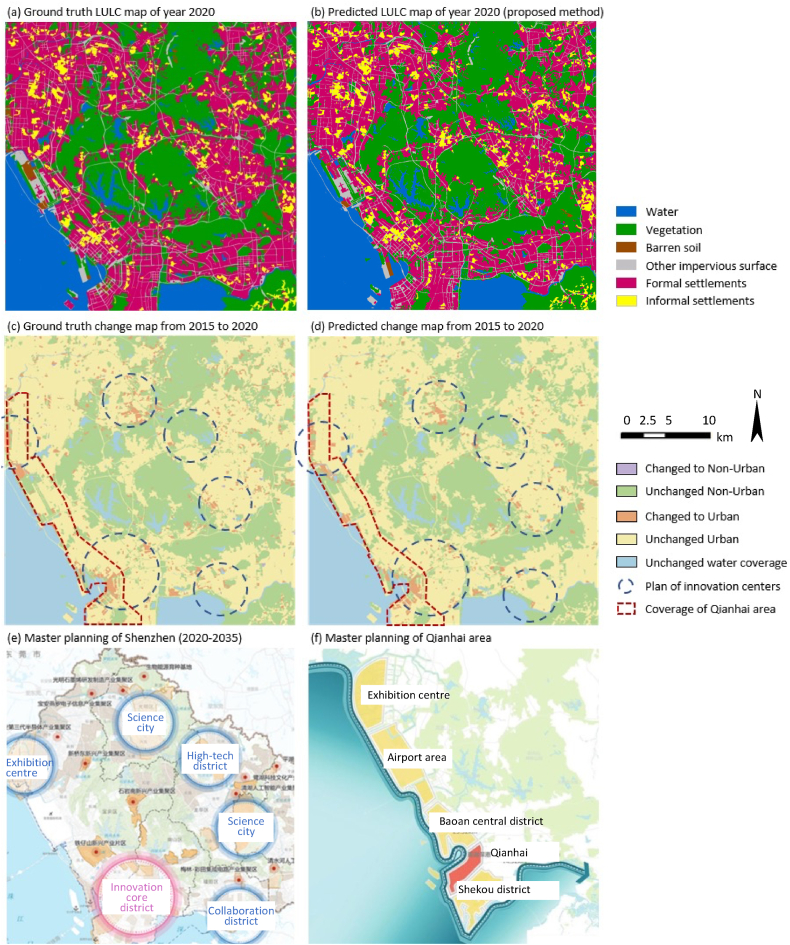
Visual inspection of the simulated results (a) the target LULC map of year 2020; (b) simulated LULC map of the proposed method; (c) target change map from 2015 to 2020; (d) simulated change map from 2015 to 2020; (e) Master planning of Shenzhen, (f) Master planning of Qianhai district.

Fig. 7 (b) depicts the simulated LULC map for the year 2020 generated by the proposed methods. To inspect the differences between the simulated LULC map and validation labels, each of them was compared with the LULC map of year 2015 for the computation of change maps. Fig. 7 (d) illustrate the simulated change map, and projected newly expanded urban areas are highlighted in orange color. It can be observed that the actual urban expansion between 2015 and 2020 mostly took place in the outskirts (e.g., along the sea and near hills), and most of these expansions were correctly predicted by the proposed method. For instance, the urban development in the harbor area of Qianhai district was projected by the proposed method. However, it still can be detected that there were some disagreements between the simulation and the target, for example, the simulated result showed false expansion around the airport area.

To compare the simulated urban expansion with the master planning of Shenzhen, Fig. 7 (e) marks the locations of six innovation centers planned in the Territorial Spatial Master Planning of Shenzhen (2020–2035), published by the Planning and Natural Resources Bureau of Shenzhen Municipal People's Government. In Fig. 7 (c) and (d), the locations of these six innovation centers are delineated with blue dashed circles in the simulated and the target change maps. Fig. 7 (f) presents the spatial coverage of the core area of the Qianhai Cooperation Zone, which is highlighted in a red polygon. In 2021, China announced to significantly enlarge the Qianhai Cooperation Zone from 14.92 km^2^ to 120.56 km^2^, which is depicted as the total areas marked with yellow and red in Fig. 7 (f). The outline of the coverage of the enlarged Qianhai Cooperation Zone is marked with red dashed lines in Fig. 7 (c) and (d) for comparison.

It can be observed that most of the actual urban expansion in these innovation centers was anticipated in the simulated results, especially for the innovation center in Northern Shenzhen. Regarding the urban development in Qianhai Cooperation Zone, the changes in the core area (red polygon in Fig. 7 (f)) were also effectively projected by the proposed method. However, the simulated result anticipated more urban expansion within the enlarged Qianhai Cooperation Zone (yellow polygons in Fig. 7 (f)), which was not yet reflected in validation target.

It is also noteworthy that although the simulated map was processed in small patches for decreasing computational cost, the heterogeneous rates of expansion still can be clearly observed in the whole study area. To be more specific, the city center areas presented more pixels that changed to urban, whereas peri-urban areas, especially the northeast region in the study area, showed substantially less expansion. This indicates that the proposed method can capture the spatio-temporal heterogeneity of land changes at scale.

### 4.2 Quantitative assessment of accuracy of the simulation

#### 4.2.1 Assessment of spatial distribution of correctness and errors (SDCE) with baseline methods

The performances of different simulation models were compared by the map comparison method of SDCE concerning the correctness of changed pixels. Since SDCE evaluation can only be conducted with binary maps, the coverage of urban extents was extracted from the six-class land use maps as binary classification maps for SDCE evaluation.

**Fig. 8:**
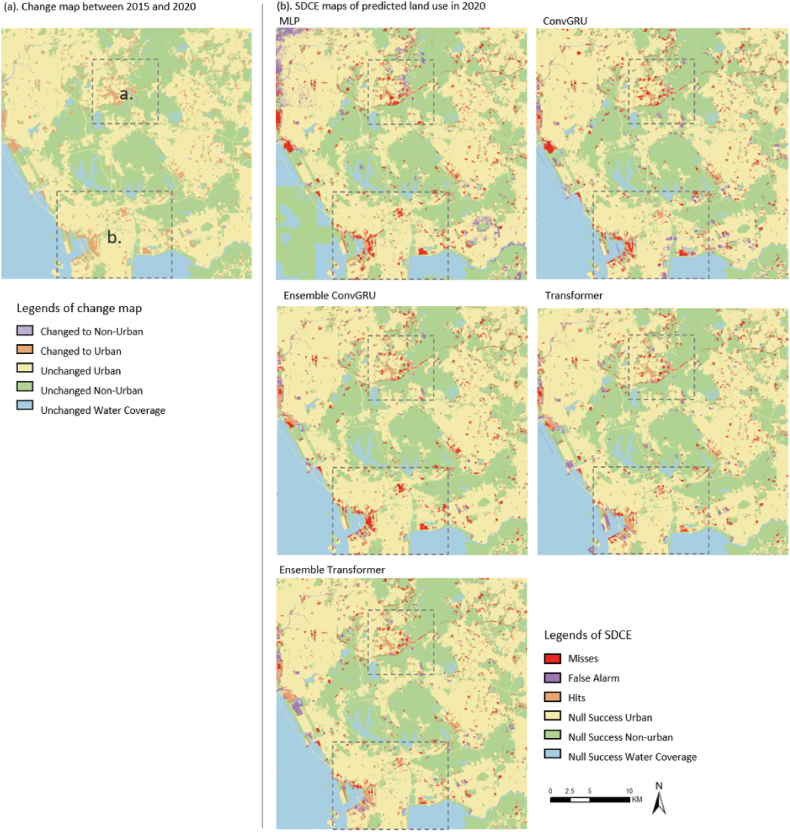
Spatial distribution correctness and errors (SDCE) produced by all the baseline methods with all the available variables.

Fig. 8 (a) shows the validation target of the change map from 2015 to 2020, in which two rectangles highlighted two areas that feature relatively substantial concentrated growth for comparison. The same locations are also marked in all the simulated SDCE maps for efficient comparison. Fig. 8 (b) shows the SDCE maps generated based on the simulated urban expansion in 2020 by all the tested methods with all the available variables. The advantage of SDCE maps is that they can directly reflect significant spatial disagreement of the changed areas between the target maps and the simulated results. As shown in Fig. 8 (b), Ensemble Transformer showed substantially more overall hits and fewer overall misses, especially in the two sub-areas (a) and (b) (i.e., areas marked with dashed squares in the target change map). Among all the baseline methods, the simulated results of MLP methods showed the worst performance in the SDCE map with large areas of misses and false alarms. In contrast, transformer-based methods (i.e., vision transformer and Ensemble Transformer) showed better overall performance than Convolutional GRU-based methods in terms of SDCE maps.

**Fig. 9:**
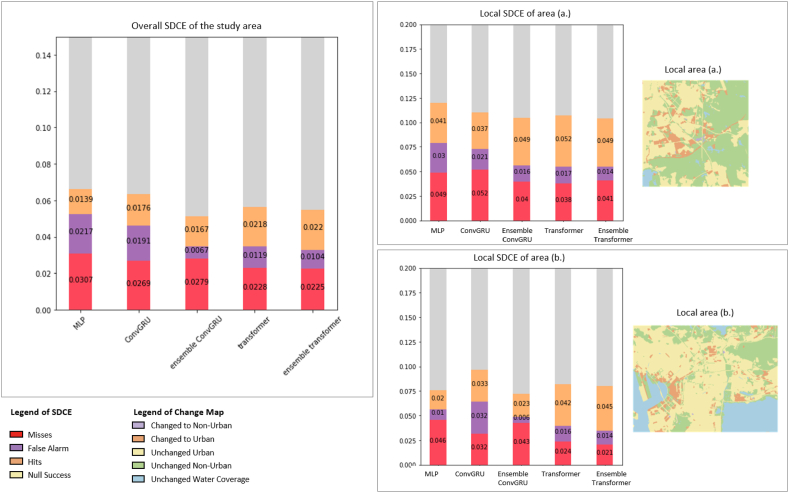
Overall SDCE and local SDCE of area (a) and (b) of all the tested methods with full variables.

The proportions of the pixels in misses, false alarms, hits, and null success are illustrated in Fig. 9 for statistical comparisons. As shown, among all the tested methods, the proposed methods yielded the largest proportion of hits, which is 2.2%. Also, the Ensemble Transformer presented the smallest percentage of misses (2.25%). Regarding false alarms, although the performance of the Ensemble Transformer did not yield the best performance, the simulated false alarm remained in a relatively small proportion (1.04%). In contrast, the ConvGRU ensemble achieved the lowest rate of false alarms, likely because it predicted fewer pixels transitioning to urban areas. This is reflected in the combined total of false alarms and hits being lower than that observed with the Ensemble Transformer. As a result, the ConvGRU ensemble not only had a smaller proportion of false alarms but also a noticeably lower rate of hits. In general, the overall SDCE evaluation underscores the superior performance of the proposed method compared to the other methods tested.

In terms of the performance in local areas, we examined two small areas in detail. Local area (a) is a scarcely built suburban area in the north of the tested area, and local area (b) is a part of the densely built-up city center in the south. For the SDCE of area (a), the proposed method achieved very close performance with Ensemble ConvGRU and transformer in terms of hits. Beyond, the proposed method showed the smallest proportion of false alarm. As for the SDCE of area (b), the Ensemble Transformer yielded the best performance in the proportions of hits (4.5%) and the proportion of misses (2.1%), the transformer achieved the second-best performance with 4.2% in hits and 2.4% in misses gained. In terms of the false alarm in area (b), transformer-based methods maintained a small proportion of false alarms. By and large, the proposed method showed the best performance in SDCE.

#### 4.2.2 Assessment of overall accuracy with baseline methods

**Fig. 10:**
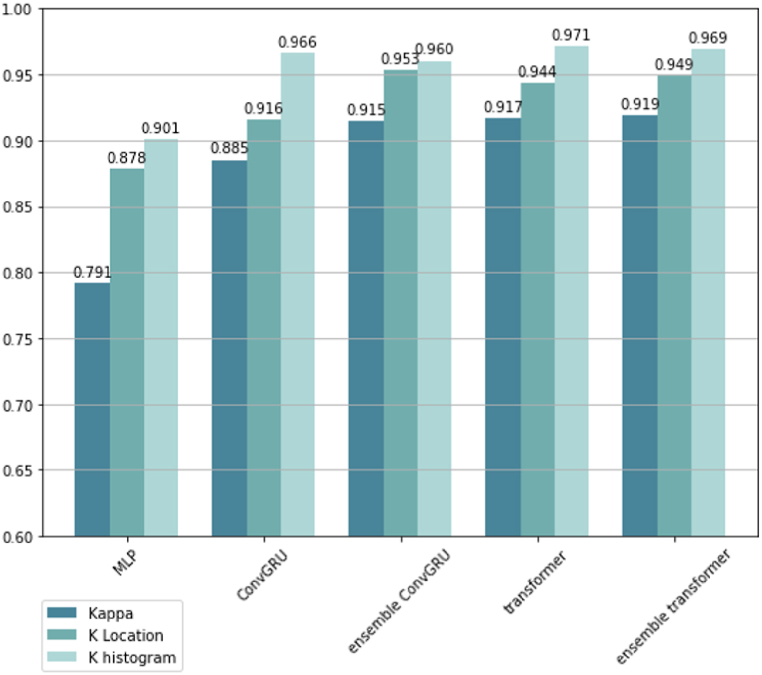
Kappa statistics of all the tested methods.

The simulated land use maps of the year 2020 by all the tested methods were also assessed by Kappa statistics, including a standard kappa, $K_{location}$, and $K_{histogram}$ (Fig. 10). All the DL methods adopted in this study, except MLP, achieved kappa coefficients above 0.88, which indicates a very high level of simulation accuracy. As can be observed, the ensemble framework brought performance gains to the original backbone models, the kappa coefficients of ConvGRU-based methods improved from 0.885 to 0.915, and the kappa coefficients of transformer-based methods increased from 0.917 to 0.919 after the implementation of the ensemble framework.

The values of $K_{location}$ and $K_{histogram}$ further reflected the performance of these tested methods in terms of location disagreement and quantification disagreement. Although Ensemble ConvGRU and transformer yielded the highest values in $K_{location}$ and $K_{histogram}$ respectively, the $K_{location}$ and $K_{histogram}$ values of the Ensemble Transformer showed a more balanced distribution than the values of Ensemble ConvGRU and transformer. The distinct distances between the values of $K_{location}$ and $K_{histogram}$ in ConvGRU method indicated its simulation result contained more location errors than the proposed method.

**Fig. 11:**
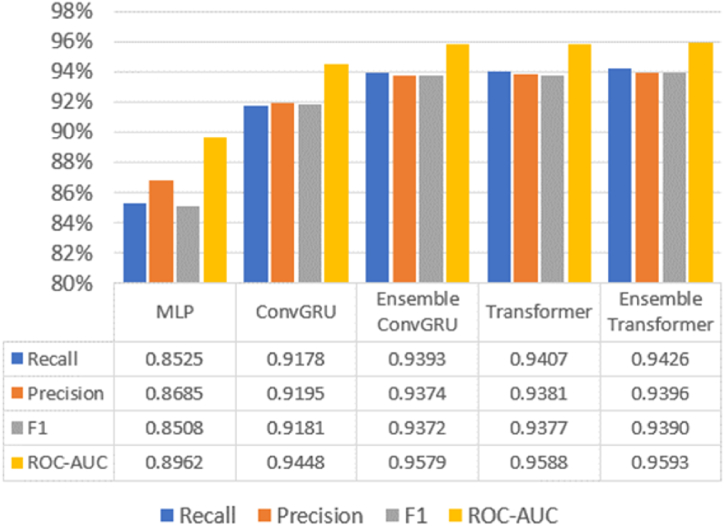
Recall, Precision, F1, ROC-AUC of all the tested methods.

All the tested models was also evaluated with Recall, Precision, F1, and ROC-AUC scores (Fig. 11). The assessment results of these metrics showed a similar pattern to the scores of Kappa statistics. Among all the tested models, the proposed method yielded the highest values across all these four evaluation metrics (Recall 94.26%, Precision 93.96%, F1 score 93.90%, and ROC-AUC score 95.93%).

**Fig. 12:**
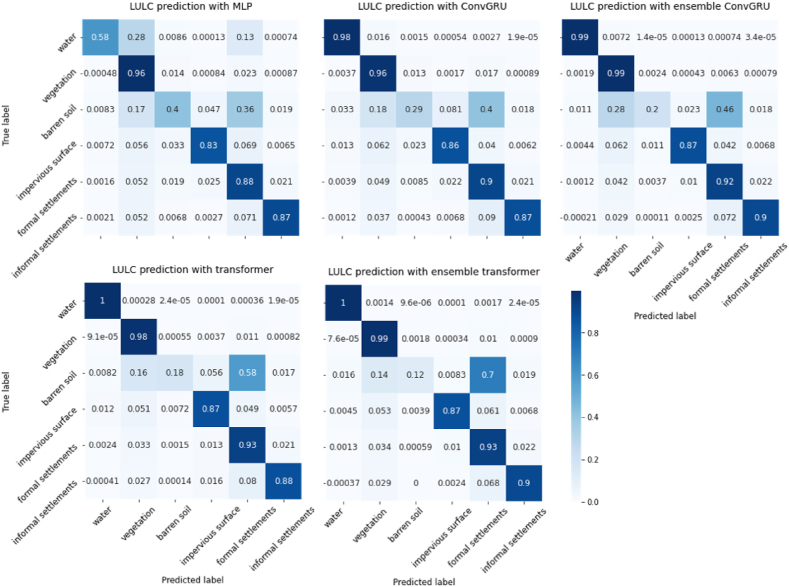
Normalized confusion matrix of all the tested prediction methods.

Fig. 12 presents the normalized confusion matrixes of the simulated results generated by different simulation models. As can be observed, Ensemble Transformer achieved the largest proportions of true positives in all LULC classes, except the class of barren soil. However, these accounted only for a small proportion of the total land use. All the tested methods tend to mistakenly predict barren soil pixels into formal settlements. This could be attribute to the imbalanced data distribution between these two classes. It also implies that the transition rules learnt from data tend to covert urban land from barren soil.

#### 4.2.3 Assessment of quantity disagreement and allocation disagreement (QDAD) of all tested methods

**Fig. 13:**
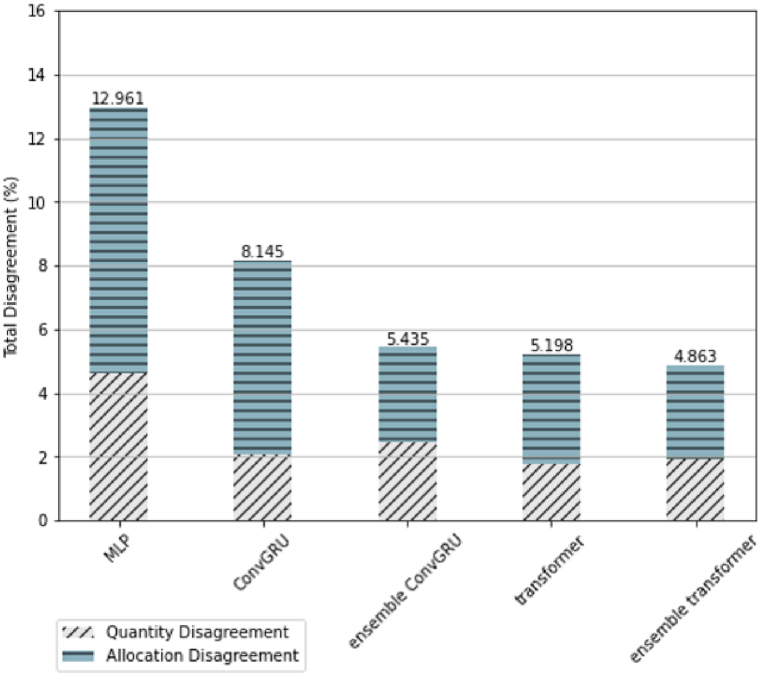
Overall QDAD of tested methods.

The assessment results of overall QDAD are presented in Fig. 13. The number marked above each bar indicates the total value of QDAD, including both quantity and allocation disagreements. Regarding the value of total disagreement, of the proposed method, the Ensemble Transformer showed the best performance with the lowest total disagreement of 4.863%. The values of the total QDAD of the Ensemble ConvGRU and transformer were 5.435% and 5.198%, respectively. The simulated result of ConvGRU yielded a substantially higher QDAD value, and the performance of MLP was the highest among all. It is noteworthy that all the tested methods showed more allocation disagreement than quantity disagreement. The balance between the two types of disagreement was relatively better in the results of the proposed method.

**Fig. 14:**
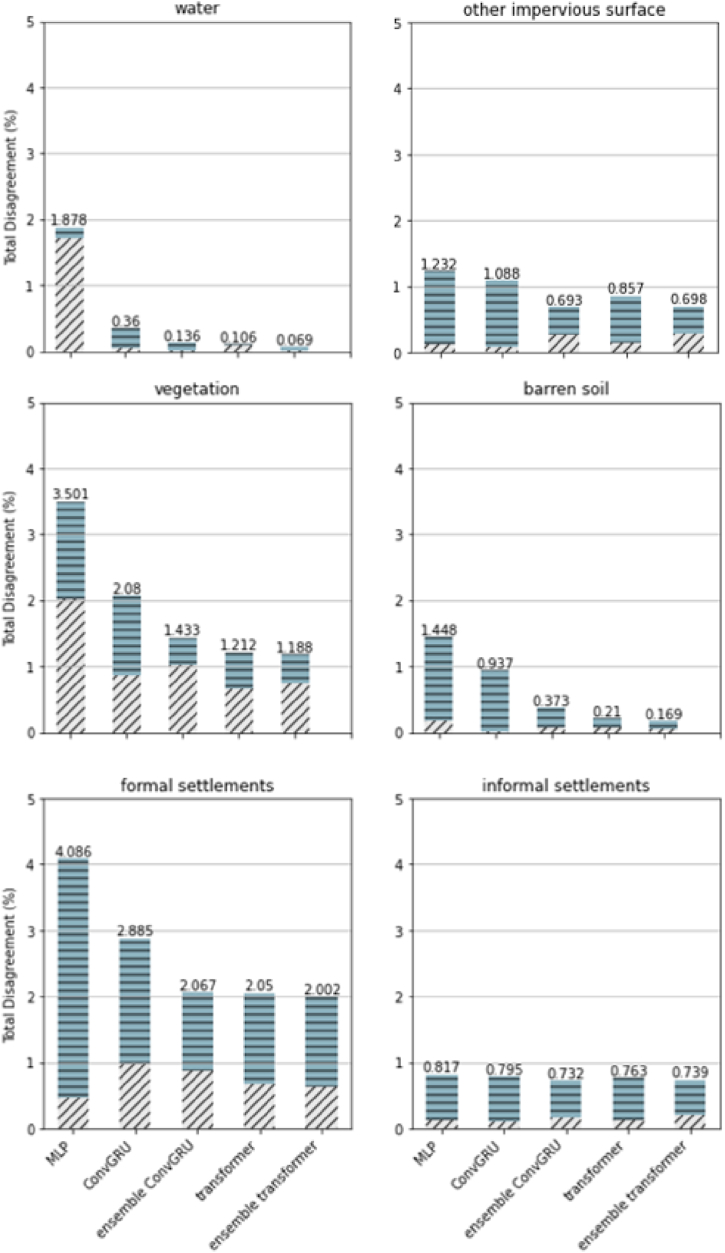
Per-class QDAD of tested methods with full variables.

For a more comprehensive evaluation, the QDAD assessments were also conducted on each LULC class (Fig. 14), which can be regarded as a breakdown of the overall QDAD. Regarding the per-class QDAD, the Ensemble Transformer achieved the lowest disagreement in water, vegetation, barren soil, and formal settlements. Although the Ensemble Transformer only gained the second-lowest disagreement in terms of informal settlements, the value of disagreement as urban extents (total disagreement of formal and informal settlement) was still the lowest among all the test methods.

### 4.3 Comparative analysis of landscape indices

The performance of the tested methods was also evaluated with five landscape metrics. Besides the simulated urban extent of year 2020, the patterns of urban extent of year 2015 and 2020 were also employed to map the trajectory of changes from the perspective of landscape indices. In this manner, a comparison regarding the consistency of simulated urban landscape patterns can be evaluated.

**Fig. 15:**
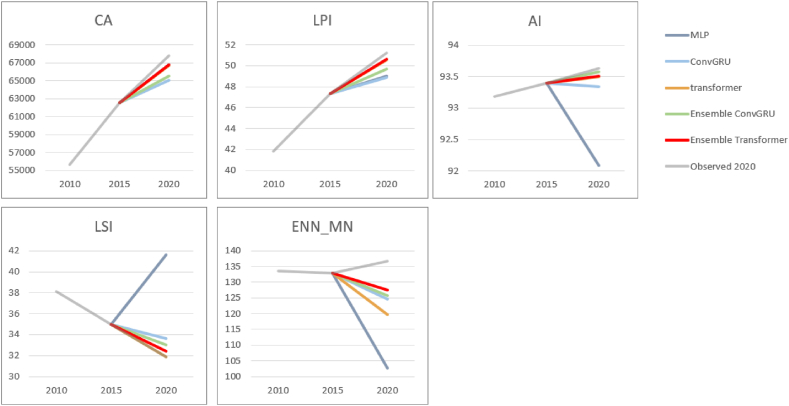
Comparison of tested methods with landscape metrics.

The simulated changes of landscape indices with all the tested DL methods are presented in Fig. 15. As can be observed, the simulation result produced by MLP generally showed the largest discrepancy compared to other methods. In contrast, Ensemble Transformer and transformer showed very similar trends with the corresponding LULC classification maps. Specifically, the Ensemble Transformer approach showed the best performance in CA, LPI, and ENN_MN. Whereas, a simple transformer yielded the smallest distances in LSI and AI, in which the Ensemble Transformer approach gained the second closed distances. This suggests that transformer-based models exhibited superior performance in achieving consistent landscape patterns.

### 4.4 Comparison with Cellerla automata (CA) method

The comparison with the SLEUTH model adopted the same settings. Specifically, the input variables of training an Ensemble Transformer model were adjusted to align with the required variables in a SLEUTH model, which include land cover, slope, urban extent, transportation, and exclusion. It should be noted that the exclusion variable employed in this experiment was the spatial coverage of water bodies.

**Fig. 16:**
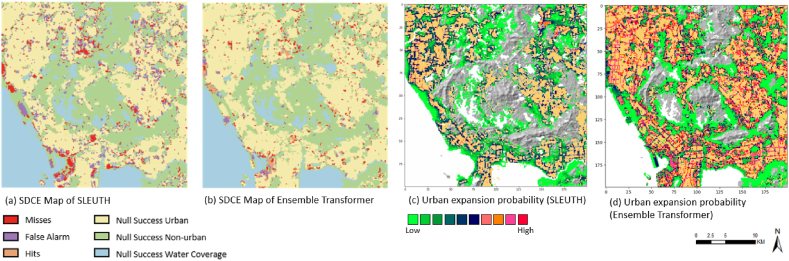
Comparison with CA method (a) SDCE map of the simulated result generated by SLEUTH, (b) SDCE map of the simulated result generated by ensemble transformer, (c) the expansion probability produced by SLEUTH, and (d) the expansion probability produced by ensemble transformer.

The simulated LULC maps with these two methods were evaluated with a series of accuracy metrics (Table 1). The proposed method yielded significantly higher accuracy compared to SLEUTH. The differences in their performance can also be detected in Fig. 16. The result simulated by SLEUTH showed a substantially larger proportion of misses and false alarms, and its probability map also indicates less likelihood of expansion in this area (Fig. 16 (c)). In contrast, the simulated map of the proposed method showed spatio-temporal heterogeneous patterns regarding the distribution of high urban expansion probability (Fig. 16 (d)). The southern downtown area exhibited a higher probability of urban expansion compared to the northern suburb area. The comparison result in this section suggests that the proposed method shows better capability in predicting incremental land changes over a relatively shorter period of time.

**Table 1:** Accuracy comparison between the simulated land change maps of year 2020 produced by the proposed method and SLEUTH model.

	Recall	Precision	F1	ROC-AUC	Kappa	K Location	K histogram	Hit/FoM (%)	Misses (%)	False Alarm (%)
**SLEUTH**	0.8342	0.8498	0.8393	0.8907	0.757	0.825	0.917	0.93%	3.45%	6.51%
**Ensemble Transformer**	0.9426	0.9396	0.939	0.9593	0.919	0.949	0.969	2.20%	1.00%	2.30%

### 4.5 Interpretation of the simulation framework

**Fig. 17:**
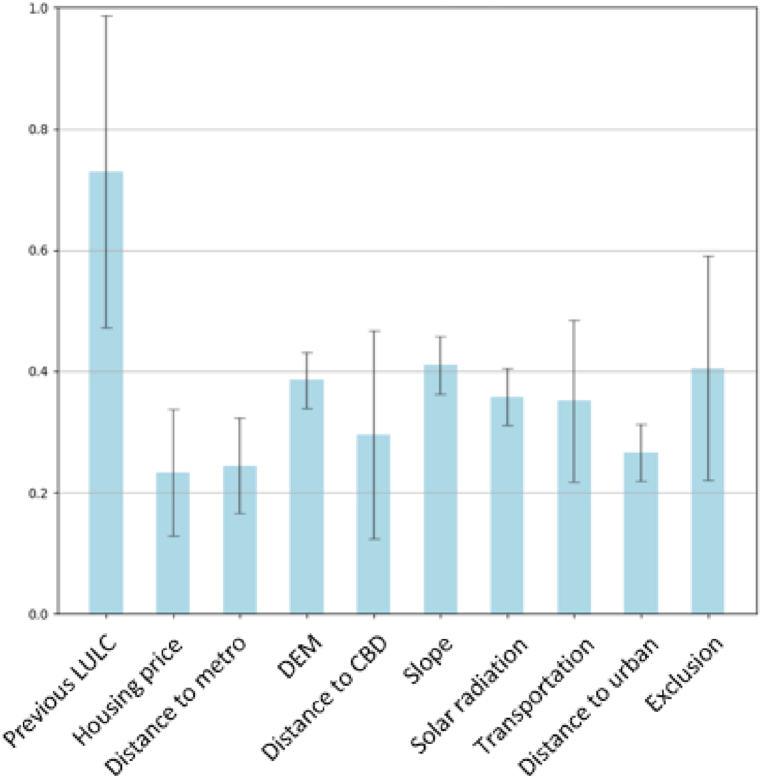
Feature importance indication generated by the channel attention weights of an Ensemble Transformer.

The proposed ensemble framework is designed for indicating feature importance through the weights of its channel attention block. We extracted the channel weights of the Ensemble Transformer trained with all the available input variables (Fig. 17). The weight of each channel reflected the importance of the corresponding encoded variable for simulation results. This mechanism aims to provide a human-interpretable approach for gaining confidence in the simulated results through examining the rationality of the feature importance of input variables.

The attention weights produced with each variable set are summarized in Fig. 17. In general, the distribution of feature importance values coincides with the variable required in the SLEUTH method. Specifically, the variables required in SLEUTH models (e.g., slope, land use, exclusion) all achieved relatively higher attention values compared to others (e.g., housing price, distance to metro). It is noteworthy that, among all the input variables, previous LULC maps gained the largest attention weights, suggesting it is the most important variable in the generation of the simulation results compared to other variables. This follows the path-dependency theory and the first law of geography, knowing where growth has previously taken place has the highest explanation value for simulated changes.

### 4.6 Simulation of future urban expansion with the proposed method

**Fig. 18:**
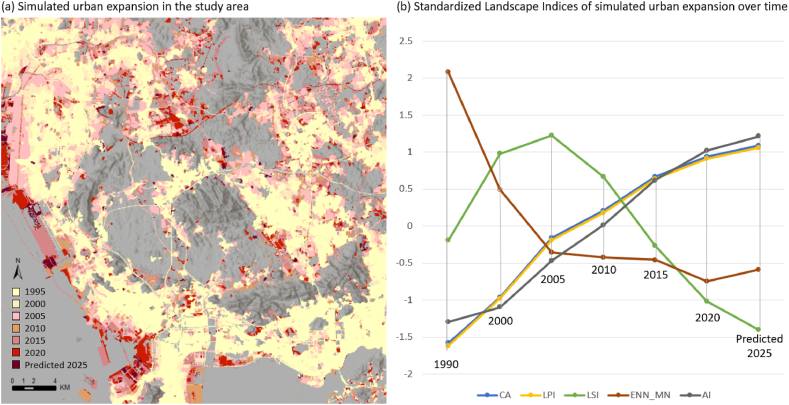
Simulated LULC map of year 2025 with the proposed method.

The urban expansion for the year 2025 was predicted with an Ensemble Transformer trained in previous sections. In Fig. 18 (a), the predicted urban extent of the year 2025 is stacked with historical urban extent maps from 1995 to 2020 for visual inspection. As the buildable land in Shenzhen became increasingly limited due to topography and local land policy, urban growth witnessed a transition from rapid spatial expansion to incremental expansion since 2010. As can be observed in Fig. 18 (a), the predicted expansion in 2025 is mainly the spatial extension of the urban edges in 2020.

An incremental and consistent growth pattern can be observed in the landscape indices depicted in Fig. 18 (b), where most of the indices appear to follow their previous trajectory for the simulated urban expansion in 2025. For instance, the AI, LPI, and CA indices of the predicted urban extent in 2025 all demonstrate a continuation of previous increasing trends, they collectively suggest a pattern of urban consolidation, where development is focused on densifying and expanding existing urban areas rather than creating new, separate urban centers. Conversely, the LSI index, which reflects the complexity of urban patch shapes, displays a substantial decline following its significant fluctuations in earlier years. The forecasted decrease in 2025 is in line with the trend of reducing complexity in urban forms as patches merge and boundaries become smoother. Furthermore, the ENN_MN index continues on its recent trajectory that started in 2005, showing slight fluctuations but an overall decrease in the average distance between urban patches. This supports the trend indicated by other indices, suggesting that urban growth is increasingly geared towards densifying and integrating existing urban spaces, thereby indicating that the predominant form of urban expansion expected in 2025 is likely to continue as infill.

## 5 Discussion

The experimental results suggest that the proposed method, the Ensemble Transformer, is capable of capturing the trajectory of urban expansion in a time-series dataset. The simulated urban expansion shows very similar patterns compared to the actual master planning of Shenzhen. Notably, the simulated tendency of urban expansion in the Qianhai Cooperation Zone aligns with the newly updated development policy in Shenzhen. This indicates that the current urban planning in Shenzhen is in line with the trajectory of its previous urban expansion patterns, and the urban areas planned for development are primarily located near the current fast-growing urban zones.

Compared to DL-based baseline methods, the proposed method demonstrated overall higher accuracy in simulating land transitioning from non-urban to urban areas. The increased accuracy of the proposed method can be attributed to the ensemble framework and the backbone transformer model. The former enhances performance through its multiple base learners, while the latter excels in learning fine-grained temporal features. This improvement is evident when comparing the performance of the Ensemble ConvGRU against the ConvGRU, as well as the higher accuracy of the transformer compared to the ConvGRU. The combined effect of the ensemble framework and the transformer backbone significantly enhances performance across most evaluation metrics. However, it lags in a few metrics, such as achieving the second-lowest rate of “false alarms” in the SDCE assessment. This is because the ConvGRU, which recorded the smallest proportion of “false alarms”, predicted much less urban expansion overall, leading to significantly poorer performance in the remaining metrics of the SDCE assessments.

To compare with CA-based methods, SLEUTH was adopted as a benchmark in this study. The significantly better performance of the proposed method is likely attributable to the scalability and adaptability of DL-based methods, which excel at processing fine-grained spatial resolution datasets and are capable of learning intricate and bespoke features from such datasets. This ability may lead to substantially better performance in predicting scenarios of incremental urban expansion.

The performance in capturing trajectory features is illustrated by an assessment using landscape metrics, which suggests that the urban expansion patterns simulated by the proposed method are more consistent in terms of urban morphology. Also, the increasing CA, LPI, and AI indicate that the predicted LULC for the year 2020 consistently features dominant and well-aggregated urban patches. The decreasing LSI and ENN-MN support this by suggesting that these patches have simpler shapes and are closer together, further indicating a tendency for less fragmented urban structure during these two decades. This tendency is also consistently reflected in the predicted LULC patterns by the proposed method for the year 2025. Arguably, the ability to capture trajectory features marks the proposed method as a novel type of simulation, demonstrating how LULC would evolve in the absence of major policy changes in the city. This provides an early warning for land use planners and complements current “what-if” modelling.

To address the lack of interpretability in DL networks, the proposed approach incorporates a channel attention mechanism to reveal the importance of each predictor variable in generating simulated urban expansion. While the proposed method can effectively highlight the significance of input variables, it does not provide explicit explanations regarding causal relationships. Arguably, the primary objective of the interpretation mechanism in this study is to establish trust and confidence in the simulation outcomes of DL methods, rather than to infer causal relationships.

There are several limitations to the proposed methodology, which primarily include the following three aspects: (i) The study focused on spatial urban land change in the horizontal dimension, which limits its ability to simulate urban renewal and vertical densification scenarios. Despite incorporating economic factors, such as housing prices, the model does not capture the full spectrum of social and economic facets of urban growth; (ii) While the proposed model outperforms conventional rule-based methods with its higher spatial resolution, capacity to leverage temporal dependencies, and reduced subjective bias, it primarily makes predictions based on learned data patterns. This approach makes deterministic control challenging, resulting in a shortfall in the ability to simulate controlled urban expansion rates for scenario planning, an area where rule-based methods excel; (iii) The proposed methodology focuses on studying the evolution of urban expansion, requiring the processing of spatio-temporal data. However, collecting time series predictor variables that align along both temporal and spatial dimensions can be challenging. It is anticipated that inconsistencies in the spatial resolutions of different predictor variables or insufficient time frames might lead to a decrease in prediction accuracy.

Future work could explore the possibility of including other types of variables in the modelling process, such as integrating socio-economic growth indicators as input variables. The generalization capability of the proposed method can also be examined with different study areas. Additionally, to apply the proposed model to scenario planning with specific growth rates, further study can test the approach of changing the momentum of the input sequence to enable the simulation of preferred growth scenarios. As discussed in the previous section, the proposed prediction method can directly indicate feature importance but not a causal relationship. Thus, an important aspect of further work could be to investigate the effectiveness of the ever-developing interpretable mechanisms for DL methods, as well as their suitability for LULC prediction tasks.

## 6 Conclusion

This study proposes a DL-based framework, Ensemble Transformer, for urban expansion simulation using spatio-temporal datasets. It can project spatio-temporal heterogeneous changes in urban expansion patterns, revealing how urban land patterns would evolve without major policy intervention in the city. To the best of our knowledge, this is the first endeavor to implement transformer-based methods in the context of urban expansion simulation. Shenzhen was chosen as the study area due to its rapid urbanization process over the past four decades.

The experiment results suggested that the proposed transformer-based methods within an ensemble framework can exhibit superior simulation capability, outperforming CNN-based methods and CA-based methods. Besides the superior performance in simulation accuracy, the proposed methods incorporate a channel-attention mechanism that can reveal the importance of features learned by the models, assisting in examining the reliability of the trained model. As indicated by the attention weights, multi-temporal land use maps are the most significant contributor to the simulated changes. Other relatively important variables for simulation include DEM, slope, and exclusion areas.

Furthermore, the proposed framework has the potential to be implemented for various spatio-temporal pattern simulations, providing critical supportive information for policymaking toward more sustainable urban growth. Although the proposed method is tailored for studying spatio-temporal LULC patterns, it has great potential for generalization and application in other studies involving time series geospatial analysis.

## Data availability statement

Data will be made available on request.

## CRediT authorship contribution statement

**Yue Zhu:** Conceptualization, Data curation, Formal analysis, Investigation, Methodology, Project administration, Software, Validation, Visualization, Writing – original draft, Writing – review & editing. **Christian Geiß:** Supervision, Writing – review & editing. **Emily So:** Supervision, Writing – review & editing. **Ronita Bardhan:** Writing – review & editing. **Hannes Taubenböck:** Writing – review & editing. **Ying Jin:** Supervision, Writing – review & editing, Conceptualization.

## Declaration of competing interest

The authors declare that they have no known competing financial interests or personal relationships that could have appeared to influence the work reported in this paper.
